# Dielectric Study of Tetraalkylammonium and Tetraalkylphosphonium Levulinate Ionic Liquids

**DOI:** 10.3390/ijms23105642

**Published:** 2022-05-18

**Authors:** Sotiria Kripotou, Georgios Tsonos, Andrea Mezzetta, Angelica Mero, Lorenzo Guazzelli, Konstantinos Moutzouris, Ilias Stavrakas, Christos Tsonos

**Affiliations:** 1 Laboratory of Electronic Devices and Materials, Department of Electrical and Electronics Engineering, University of West Attica, 12244 Athens, Greece; kroula@gmail.com (S.K.); gtsonos@gmail.com (G.T.); moutzouris@uniwa.gr (K.M.); ilias@uniwa.gr (I.S.); 2 Department of Pharmacy, University of Pisa, Via Bonanno 6, 56126 Pisa, Italy; andrea.mezzetta@for.unipi.it (A.M.); angelica.mero@phd.unipi.it (A.M.); lorenzo.guazzelli@unipi.it (L.G.); 3 Department of Physics, University of Thessaly, 35100 Lamia, Greece

**Keywords:** ionic liquids, electrical conductivity, dielectric properties

## Abstract

Broadband dielectric spectroscopy in a broad temperature range was employed to study ionic conductivity and dynamics in tetraalkylammonium- and tetraalkylphosphonium-based ionic liquids (ILs) having levulinate as a common anion. Combining data for ionic conductivity with data obtained for viscosity in a Walden plot, we show that ionic conductivity is controlled by viscosity while a strong association of ions takes place. Higher values for ionic conductivities in a broad temperature range were found for the tetraalkylphosphonium-based IL compared to its ammonium homolog in accordance with its lower viscosity. Levulinate used in the present study as anion was found to interact and associate stronger with the cations forming ion-pairs or other complexes compared to the NTf_2_ anion studied in literature. In order to analyze dielectric data, different fitting approaches were employed. The original random barrier model cannot well describe the conductivity especially at the higher frequencies region. In electric modulus representation, two overlapping mechanisms contribute to the broad low frequencies peak. The slower process is related to the conduction mechanism and the faster to the main polarization process of the complex dielectric permittivity representation. The correlation of the characteristic time scales of the previous relaxation processes was discussed in terms of ionic interactions.

## 1. Introduction

Research into ionic liquids (ILs) continues with undiminished interest due to the possibility of customizing their physicochemical properties to suit specific applications by systematic variations of the constituent ions; thus, they are referred to in the literature as “designer solvents”. A particularly interesting sub-set of ILs are those based on tetraalkylammonium and tetraalkylphosphonium cations, which often display remarkable electrochemical windows and high hydrophobicity, when alkyl chains of suitable lengths are present. Changing the central atom of cation from nitrogen to phosphorus, i.e., tetraalkylammonium versus tetraalkylphosphonium has a marked effect upon the physicochemical properties of these ILs. Indeed, ILs based on the tetraalkylphosphonium cation exhibit significantly reduced viscosities [1,2,3,4,5], enhanced ionic conductivities [1,2,5], as well as higher thermal degradation temperatures relative to their ammonium homologs [1]. In the IL research arena, a clear tendency has emerged in recent years, namely the replacement of ions obtained from fossil fuel-derived materials with bio-based ions prepared from renewable sources [6]. In this context, polysaccharides, such as cellulose and hemicellulose, are considered valuable sources of compounds for the development of anion bio-based ILs. Indeed, sugars can be easily converted into smaller molecules i.e., formic acid, acetic acid, levulinic acid, furfural, and hydroxymethyl furfural [7,8]. Besides formate and acetate ILs, a few different levulinate ILs have been prepared to date and showed promising features in the pharmaceutical field [9], in asymmetric catalysis [10], in CO_2_ absorption processes [11,12], or in the dissolution and modification of cellulose [13,14,15,16].

Broadband dielectric spectroscopy (BDS) has been widely used to study ionic conductivity and dynamics in ILs. Ionic conductivities are directly read off from the data, while information on the dynamics is achieved by fitting dielectric data by proper equations. A relaxation process is always present in dielectric spectra of ILs; however, its origin is a matter of debate in the literature. One can find studies where this relaxation process is considered as the ion conductivity relaxation [17,18,19] and others where the relaxation process is assumed to arise from the reorientational motion of cations [20,21,22] or from correlated anion–cation motions [23]. Contributions in dielectric response from both conductivity relaxation and other relaxations have also been reported [24,25,26,27]. The origins of the additional contribution’s structural reorganization of ionic atmosphere [28], interfacial polarization [25], artificial due to insulating impurities [24,29] and reorientation of dipolar cation [27], have been proposed.

To model ion conductivity relaxation in ILs, the random barrier model (RBM) developed by Dyre in 1985 [30] and modified in 2008 [31] has been widely used. According to the RBM, in disordered materials, ion transport [32] always results in a polarization process, which is due to the ion motion within short length scales or at short time scales. At a longer time scale, the mobile ions overcome the largest energy barrier of the system and will contribute to the dc conductivity. The original RBM successfully describes the data for many ILs in conductivity formalism [17,19] but there are cases where it lacks reproduction of the data in permittivity formalism [33]. An IL ion cage (IC), which is formed by all the anions in the first solvation shell of a cation or vice versa, and an ion-pair (IP), which is defined as a partial association of oppositely charged ions to form distinct chemical species, coexist [34]. The IC concept has been used by Gainaru et al. [33] to explain the origin of the conductivity process observed in dielectric spectra. According to this model, dielectric relaxation is due to the polarization mechanism caused by ions escaping from the cage formed by surrounding counterions. On the other hand the existence of ion association in ion-pairs and more complex structures has been used to explain lower ionicity, compared to that expected according to their viscosity, observed in ILs. The positions of the data of molar conductivity versus viscosity relative to the “ideal” line in the Walden plot as described by Angell et al. [35] have been used as measures of ion association.

In this article, ILs with tetraalkylammonium and tetraalkylphosphonium as cation and levulinate, as common anion, were studied using broadband dielectric spectroscopy to determine the effect of changing the cationic central atom on ionic conductivity and the dynamics in these systems. Dielectric studies in ILs based on tetraalkylphosphonium [36,37] and tetraalkylammonium cation [25,27,38] can be found in the literature. However, there are few comparative dielectric studies of ammonium and phosphonium-based ILs [26], while no dielectric studies on levulinate-based ILs can be found, to the best of our knowledge. The ecological transition ‘requests’ to move toward renewable compounds and materials as fast as possible. However, performances of these materials should be comparable to fossil-based ones. To understand properly the pros and cons of new materials, the characterization of their physicochemical properties is of primary importance. In more detail, some of the main research areas where ILs are employed include lignocellulose biomass fractionation, dissolution, transformation, and/or functionalization. In this regard, the proposed structures showed promising solvation capability towards cellulose [15]. The present work calls for an in-depth study on the properties of ILs prior to their use in whatever application. This would help to rationalize the data available in the literature, which are often based on the trial-and-error approach. Here, it is shown that substitution of nitrogen with phosphorus results in higher values of ionic conductivity, which is controlled by the viscosity of the systems, while no effect in ion association is found. However, compared to other anions reported in the literature, levulinate associates stronger with cations. Regarding dynamics, an analysis of the dielectric response employing different fitting approaches reveals the contribution of the conductivity relaxation in electric modulus representation and the main polarization peak in complex dielectric permittivity formalism observed in ILs. The results are discussed in terms of the ionic interactions, since, with a better understanding of the relationship between structure and properties in ionic liquids, it is possible to design and manufacture materials with desired application properties.

## 2. Results and Discussion

### 2.1. Dielectric Data

**Figure 1:**
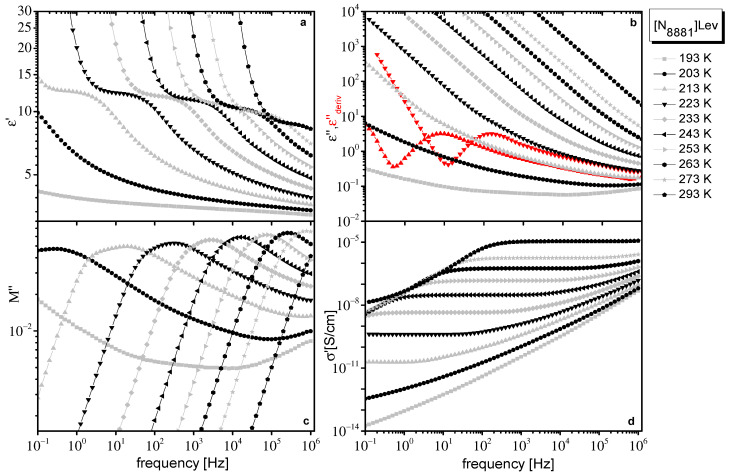
Different representations of dielectric data obtained for [N_8881_]Lev at the temperatures indicated on the plot. Real part of dielectric permittivity ε′(f) (**a**), imaginary part of dielectric permittivity, ε″(f) (**b**), derivative of ε′, $\varepsilon_{\mathrm{deriv}}^{\prime \prime }(f)$ discussed in the text (at two selected temperatures, red symbols in (**b**), imaginary part of electric modulus, M″(f) (**c**), and real part of conductivity, σ′(f) (**d**).

**Figure 2:**
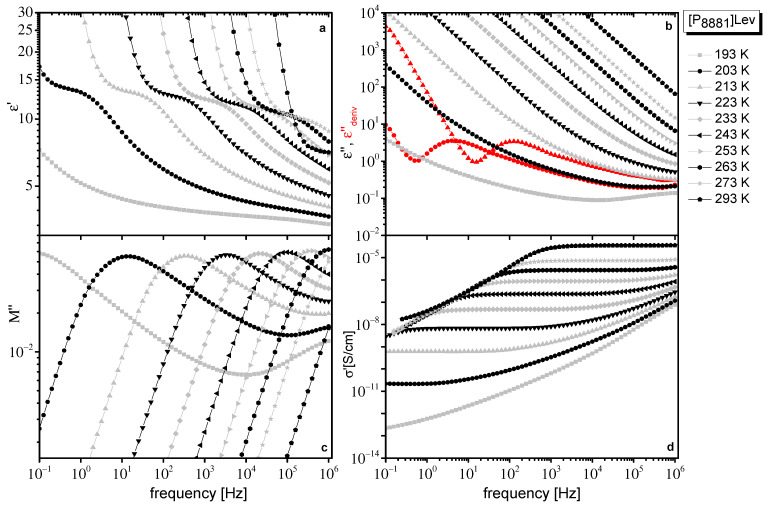
Different representations of dielectric data obtained for [P_8881_]Lev at the temperatures indicated on the plot. Real part of dielectric permittivity ε′(f) (**a**), imaginary part of dielectric permittivity, ε″(f) (**b**), derivative of ε′, $\varepsilon_{\mathrm{deriv}}^{\prime \prime }(f)$ discussed in the text (at two selected temperatures, red symbols in (**b**), imaginary part of electric modulus, M″(f) (**c**) and real part of conductivity, σ′(f) (**d**).

In Figure 1 and Figure 2, different representations of dielectric data at various temperatures obtained for [N_8881_]Lev and [P_8881_]Lev, respectively, are presented. Although quantities in different representations of dielectric data are interrelated and contain equivalent information, dielectrically active processes of ILs are differently emphasized in each representation, giving complementary information, making their separate discussions helpful.

Dielectric response of both ILs is qualitatively similar to the one found for ILs in the literature. In the real part of the complex dielectric permittivity ($\varepsilon^{*}\left(f\right)=\varepsilon^{\prime }\left(f\right)-i\varepsilon^{\prime \prime }\left(f\right))$, ε′, a step that shifts to higher frequencies with increasing temperature, which evidences a polarization process, is observed (Figure 1a and Figure 2a). In the imaginary part of dielectric permittivity, ε″, contribution of dc conductivity, ${\;\sigma }_{0}$, dominates, giving a linear dependence of ε″ with frequency, f, ($\varepsilon^{\prime \prime }=\frac{\sigma_{0}}{2{\pi \varepsilon }_{o}}f^{-1}$). The large values of ε″, at low frequencies due to dc conductivity contribution, mask any loss peaks (Figure 1b and Figure 2b). Dielectric relaxations are resolved as peaks only when the derivative of ε′, which is free from dc conductivity contribution, is used. In this approximation, also known as the “conduction free” approximation, ε″ is approximated by $\varepsilon_{\mathrm{deriv}}^{\prime \prime }=-\frac{\pi }{2}\;\frac{\partial \varepsilon^{\prime }\left(\omega \right)}{\partial \ln\omega }$ [39]. Data in the $\varepsilon_{\mathrm{der}}^{\prime \prime }$ representation are shown as red curves in Figure 1b and Figure 2b, where a peak is visible. The dc conductivity is directly evidenced by the frequency independent regions revealed in the real part of conductivity ($\sigma^{*}\left(f\right)=\sigma^{\prime }\left(f\right)+\sigma^{\prime \prime }\left(f\right)$), σ′ (Figure 1d and Figure 2d). The dc plateau in σ′ is followed by an approximately power law increase at higher frequencies, behavior that is often regarded as typical for hopping conductivity in a disordered matter.

In the imaginary part of the electric modulus, M″ ($M^{*}\left(f\right)=M^{\prime }\left(f\right)+iM^{\prime \prime }\left(f\right)=1/\varepsilon^{*}\left(f\right)$) a broad peak can be followed (Figure 1c and Figure 2c). Broadening of the peak is more pronounced at low temperatures and especially for [N_8881_]Lev where M″ reveals a two-contribution structure. In M″ representation, the low-frequency steady increase in ε″ caused by conductivity and discussed above is converted into a peak. The peak frequency, f_M_, which depends on the value of dc conductivity, σ_0_, and the dielectric constant ε_s_, $\sigma_{0}=\varepsilon_{o}\varepsilon_{s}2{\pi f}_{Μ}$ [40], is often used as the characteristic frequency of conductivity relaxation in ILs. The peak observed in M″ arising from conductivity usually occurs close to the crossover frequency between the frequency independent (dc) and the frequency dependent (ac) part of σ′. However, the polarization processes (dielectric relaxations) also lead to peaks in M″, which are shifted to higher frequencies compared to the peaks observed in ε″ [39]. So, the broad peak in M″, especially its double structure observed for the systems under investigation (Figure 1c and Figure 2c), indicates the contribution of a dielectric relaxation except the conductivity relaxation in the dielectric response of these IL systems. Moreover, the linear increase of M″ values at lower temperatures and at the higher frequency region in logM″–logf plots (Figure 1c and Figure 2c), indicates the contribution of a faster additional dielectric relaxation. The influence on the frequencies window of a high frequency dielectric relaxation can also be verified if the slope of diagrams logM″–logf is not constant at the higher frequencies region.

At low frequencies and high temperatures, dielectric response is dominated by electrode polarization (EP) effects, caused by the accumulation of immobilized mobile charge carriers at the interface between the sample material and metal electrodes [41,42,43,44]. EP effects give rise to large values of ε′ (Figure 1a and Figure 2a) and a drop in σ′ (Figure 1d and Figure 2d) at low frequencies. In ε″, the EP effect causes a deviation from f^−^^1^ dependence, while in $\varepsilon_{\mathrm{deriv}}^{\prime \prime }$, there is a power law increase at low frequencies, which leads to the formation of a peak at even lower frequencies (Figure 1b and Figure 2b). In the modulus representation, EP effects are eliminated.

### 2.2. Dielectric Data Evaluation

Dielectric data evaluation gave information on the ionic conductivity and on the dynamics of the ILs. In the following, the results regarding ionic conductivity and dynamics are discussed separately.

#### 2.2.1. Ionic Conductivity

**Figure 3:**
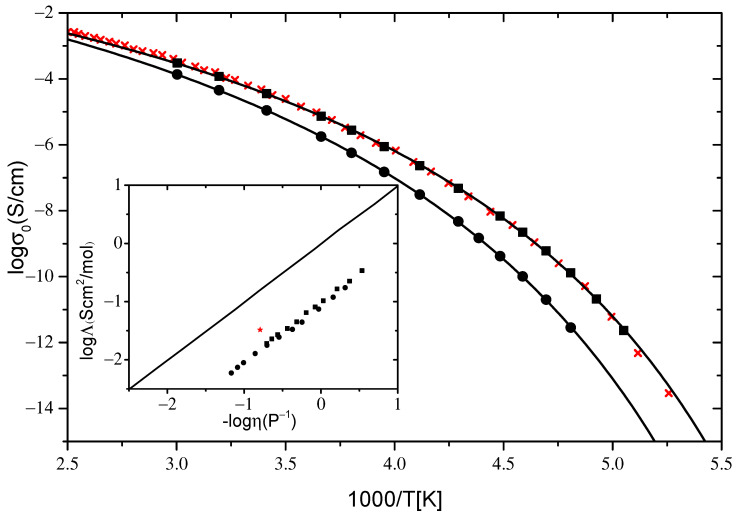
Arrhenius diagram of conductivity σ_0_ [S/cm] for [P_8881_]Lev (squares) and [N_8881_]Lev (circles) IL systems. The solid lines are VFT fits to the data. The red crosses are reproduced data from [25] for conductivity of [N_8881_]NTf_2_ IL system. The inset shows a Walden plot for the systems under investigation and the [N_8881_]NTf_2_ IL system (red star) along with the “ideal” Walden line (KCl aqueous solution).

Conductivity values were directly read off from the distinct dc plateaus in σ′(f) spectra (Figure 1d and Figure 2d) and plotted in a common Arrhenius diagram for both IL systems under investigation in Figure 3. For both IL systems, the dc conductivity reveals the typical non-Arrhenius temperature dependence known from glass forming systems. The data are well described by the empirical Vogel–Fulcher–Tammann (VFT) expression:

(1) $$\sigma =\sigma_{\infty }\exp\left(\frac{-B}{T-T_{0}}\right)$$

where $\sigma_{\infty }$, $T_{0}$, and B are fitting parameters. The value of the parameter $T_{0}$ is a few dozen lower than the glass transition temperature, $T_{g}$, and B = DT_0_, where D is the so-called strength parameter, which is used to distinguish between strong and fragile glass formers [45]. A small value of D leads to a significant deviation from the Arrhenius-type behavior and vice versa. Alternatively, the fragility index m is also used for the classification of glass formers. Both parameters are connected via the relation m≈16+590/D [46]. Fits of the VFT equation on the data are shown as solid lines in Figure 3. Slightly higher values of D and correspondingly lower values of m were found for [N_8881_]Lev compared to [P_8881_]Lev (D = 13.2, m = 61 and D = 11.7, m = 66, respectively). As pointed out [47], except for the influence of the glass transition temperature, T_g_, on the room temperature conductivity of ILs, an important role, ‘plays’ the fragility (m or D). A higher value of m found for [P_8881_]Lev compared to [N_8881_]Lev is consistent with the higher conductivity at room temperature found for it (Figure 3). Table 1 shows the VFT parameters obtained from fitting of conductivity for [N_8881_]Lev and [P_8881_]Lev.

Higher values of ionic conductivity were found for the phosphonium-based IL compared to its ammonium homolog across the full temperature range studied, in accordance with the results, regarding the effect of changing the cation charge center from nitrogen to phosphorus, previously reported [1,2,26]. The fact that the reported results refer to IL systems with cations having different lengths of alkyl chains and different anions than those studied here indicates that this is a general trend. Higher values of ionic conductivity found for [P_8881_]Lev compared to [N_8881_]Lev can be understood in terms of lower viscosity and T_g_ values found for the former [48]. In the inset of Figure 3 the logarithm of molar conductivity was plotted as a function of the logarithm of inverse viscosity, known as a Walden plot [35,49]. Molar conductivity was calculated as $\Lambda =\sigma_{0}Μ/\rho$, where M is the molecular weight and ρ the density. For the calculations, ρ values obtained for the same systems reported in [48] were used. The Walden plot was used for the determination of the relationship between ionic conductivity and viscosity as well as for a qualitative description of ILs regarding their ion association. The data for both ILs studied here fall in a straight line of slope close to unity (1.0 for [P_8881_]Lev and 0.97 for [N_8881_]Lev), indicating that the charge transport in both systems is controlled by viscosity, as has been reported for many aprotic ILs [2,49,50,51]. The charge and mass transport in the investigated ILs are well coupled to each other, pointing to a vehicular charge transport, dominating its conductivity response at least at high temperatures where measurements of both techniques are available.

The position of the Walden curve for a given system relative to the “ideal” Walden line, which corresponds to the fully dissociated electrolyte solution where ionic species are equally mobile, is often used as a qualitative measure of the iconicity of ILs. From the deviations of the curve, from the reference line in the Walden plot, Angell et al. classified specific ILs as either “good” ILs, “poor” ILs, or non-ionic (molecular) liquids [35,52]. Here, we do not use the Walden plot to classify ILs under investigation regarding their iconicity but rather to compare the results obtained for similar systems. As can be seen in the inset of Figure 3, where the “ideal” line is plotted as a solid line, data for both IL systems lie below this line, indicating strong interactions between ions and the existence of highly associated ions in these systems. Similar behavior has been reported for other tetraalkylammonium and tetraalkylphosphonium-based ILs [49]. No significant difference in ion association between ILs studied here was found.

Data for ion conductivity of [N_8881_]NTf_2_ IL reported in the literature [25] are plotted as crosses in Figure 3, along with current results. Considerably lower ion conductivity values were obtained for [N_8881_]Lev compared to [N_8881_]NTf_2_ in accordance with the higher viscosity measured for the former (at 298 K 1019 mPas [48] and 620 mPas [4], respectively). Interestingly, the same values for ionic conductivity were found for [N_8881_]NTf_2_ and [P_8881_]Lev although lower viscosity values were measured for the latter (at 298 K 620 mPas [4] and 366 mPas [48], respectively). The lower conductivity values from that expected according to its viscosity obtained for [P_8881_]Lev, could be understood in terms of enhanced ion association in this IL compared to [N_8881_]NTf_2_. Datum for [N_8881_]NTf_2_ at 298 K, using a value of ρ equal to 1.1 g/cm^3^, reported in [25], is included as red star in the Walden plot (inset of Figure 3). The positions of the data obtained for [N_8881_]NTf_2_ relative to those obtained for the ILs under investigation indicate a stronger ion association in the latter.

#### 2.2.2. Dynamics

**Figure 4:**
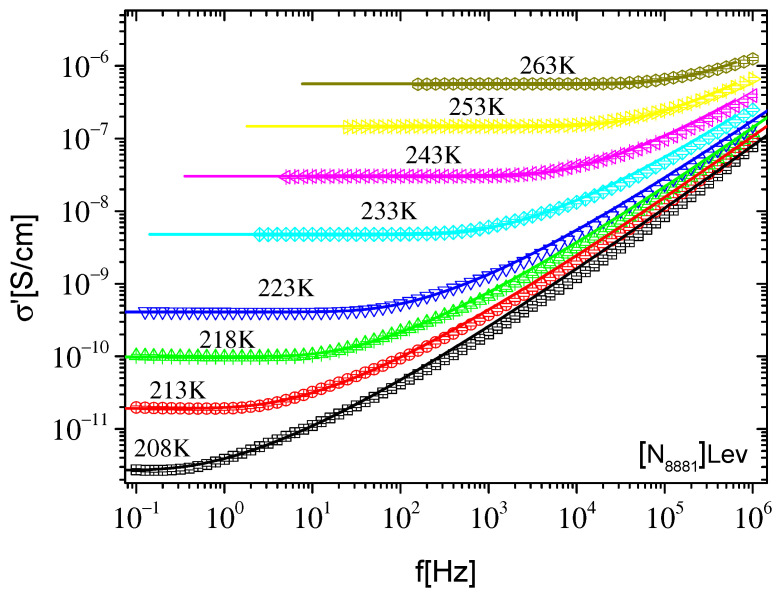
Real part of conductivity σ′ as a function of frequency f at different temperatures, indicated on the plot, for [N_8881_]Lev. The lines are best fits of the expressions of the original RBM performed in the frequency region of the dc–ac transition and extrapolated to the whole frequency range.

**Figure 5:**
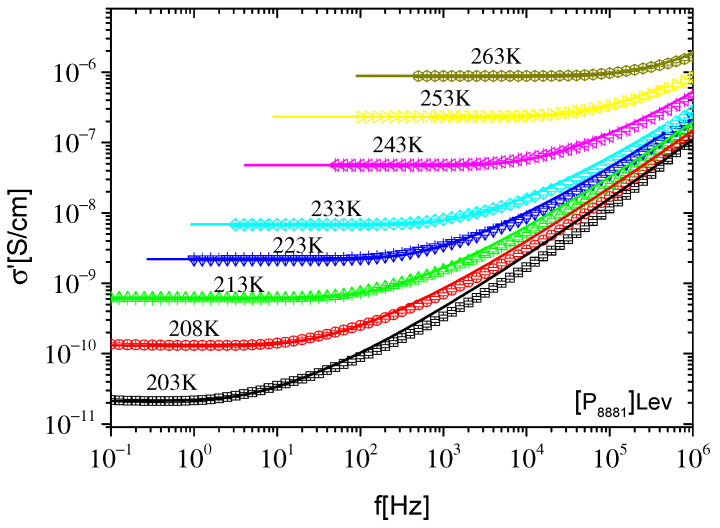
Real part of conductivity σ′ as a function of frequency f at different temperatures, indicated on the plot, for [P_8881_]Lev. The lines are best fits of the expressions of the original RBM performed in the frequency region of the dc–ac transition and extrapolated to the whole frequency range.

Dielectric data were evaluated in different representations using different model functions in order to extract information on the dynamics in ILs under investigation. The RBM model in its original version [30] as well as the phenomenological Havriliak–Negami (H–N) model [53] were employed. In the framework of the original RBM model, the expression for the complex conductivity is given by the following simplified equation:

(2) $$\sigma^{*}\left(f\right)=\sigma_{0}\left(\frac{i2{\pi f\tau }_{\mathrm{RBM}}}{\ln\left(1+i2{\pi f\tau }_{\mathrm{RBM}}\right)}\right)$$

where σ_0_ is the dc conductivity value and τ_RBM_ is a time constant at which the transition from ac to dc conductivity takes place. The time constant τ_RBM_ corresponds to the attempt rate f_RBM_ (f_RBM_ = 1/2πτ_RBM_) to overcome the highest energy barrier. For f = f_RBM_ Equation (2) gives σ′ = 1.17σ_0_. The real part of Equation (2) was used to fit σ′(f) data in the frequency region of dc–ac transition. The RBM model describes relatively well the experimental data of both ILs in the selected region, while it lacks describing the data at higher frequencies, as can be seen in Figure 4 and Figure 5. When the fit is performed in the whole frequency range (not shown here) a negative divergence of the fitting curves from experimental data are observed even in the region of the ac–dc transition. However, it should be noted here that the use of the RBM model is questionable, and is obviously not expected to describe the dielectric response of materials when the basic assumptions of this model are not met. The ILs studied here do not satisfy the assumptions and considerations of the RBM model, because in addition to the existence of mobile ions that create a polarization mechanism at low frequencies, and at even lower frequencies contribute to the dc conductivity, there is also the contribution of a faster dielectric relaxation at higher frequencies, as mentioned above. As will be seen below, the effect of this faster dielectric mechanism is significant in the frequency window of the present study. Thus, it would be expected that the discrepancy of the RBM model at high frequencies would be due to the fact that the contribution of this fast mechanism was not taken into account in Equation (2). In the case where the extension of the best fit of Equation (2) resulted in lower values compared to the experimental data in the high frequency range, the lack of the faster mechanism contribution to Equation (2) could explain the inability of this relation to describe the conductivity response at higher frequencies. However, the extensions of the best fit of Equation (2), in both ionic liquids, lead to higher values than the corresponding experimental data in the high frequencies region, as shown in Figure 4 and Figure 5. Thus, it becomes clear that the RBM model is not able to describe satisfactorily the overall conductivity response of ILs studied in the present work. The application of the original RBM in ionic liquids describes the conductivity spectra σ′(f) reasonably well but is not able to describe the data in the ε*(f) representation [33]. Similar behavior was reported in [N_8881_][NTf_2_] ionic liquid where the RBM describes the data in σ′(f) very well, but underestimates the data in σ″(f) at lower frequencies and, as a consequence, lacks describing the ε′(f) [27]. In the present study, the original RBM describes reasonably well the dc–ac region but overestimates the data in σ′(f) at higher frequencies. In the framework of RBM, noninteracting charge carriers were considered to perform hopping on a simple cubic lattice. The charge transport process was governed by a broad distribution of energy barriers while the charges have to overcome a certain percolation barrier in order for random diffusion to take place. So, the inability of RBM to describe the overall conductivity response of some ionic liquid systems is possibly due to the fact that some of the basic assumptions of this model cannot satisfactorily describe the charge transport mechanisms in these materials. It is also possible that the transport of ions is accompanied by dipolar contribution, as a result of their structures, which is not taken into account by RBM.

The fitting procedure traditionally used to analyze the dielectric data of molecular liquids was employed to describe the dielectric data obtained for the ILs under investigation. The analysis was performed in the $\varepsilon_{\mathrm{deriv}}^{\prime \prime }$ formalism using the (derived in this formalism) equation, assuming the H–N equation for the description of ε′ [39] given below:

(3) $$\varepsilon_{\mathrm{deriv}}^{\prime \prime }=-\frac{\pi }{2}\frac{\partial \varepsilon_{\mathrm{HN}}^{\prime }}{\partial \ln2\pi f}=\frac{\pi }{2}\frac{\mathrm{ab}\Delta \varepsilon {\left(2\pi \tau \right)}^{\alpha }\;\cos\left[\frac{\alpha \pi }{2}-\left(1+b\right)\theta_{\mathrm{HN}}\right]}{{\left[1+2{\left(2\pi f\tau \right)}^{\alpha }\cos\left(a\pi /2\right)+{\left(2\pi f\tau \right)}^{2\alpha }\right]}^{\left(1+b\right)/2}}$$

where $\theta_{\mathrm{HN}}=\arctan\left[\sin\left(\frac{\pi \alpha }{2}\right)/({\left(2\pi f\tau \right)}^{-a}+\cos\left(\pi \alpha /2\right))\right]$.

**Figure 6:**
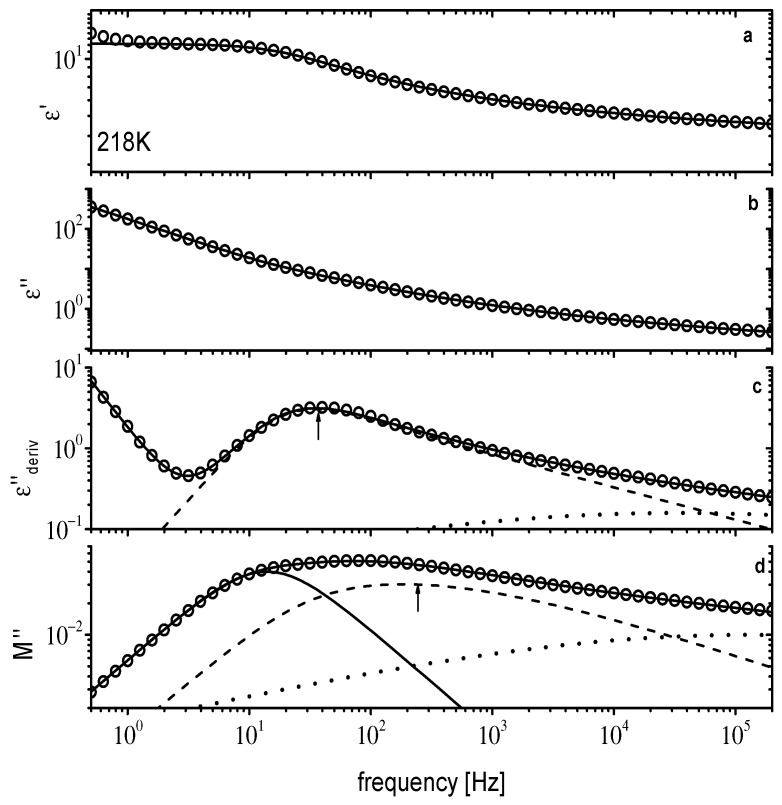
Dielectric data for [N_8881_]Lev at 218 K. ε′(f) in (**a**), ε″(f) in (**b**), $\varepsilon_{\mathrm{deriv}}^{\prime \prime }$(f) in (**c**), and M″(f) in (**d**). Solid lines are fitting curves. In (**c**,**d**) individual relaxations are shown as dashed and dotted lines. The solid line in (**d**) is conductivity relaxation (see text for details). The arrows in (**c**,**d**) indicate the main peak position.

**Figure 7:**
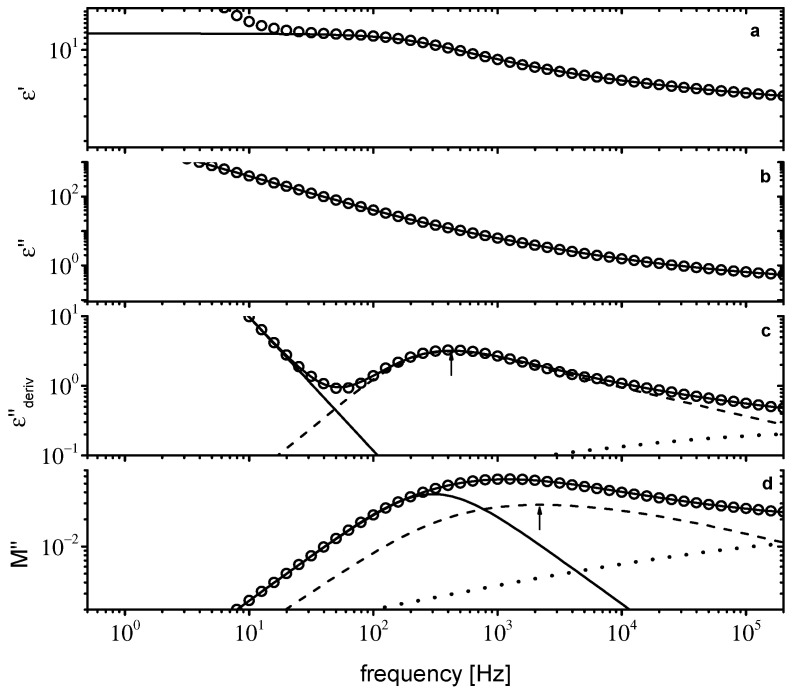
Dielectric data for [P_8881_]Lev at 218 K. ε′(f) in (**a**), ε″(f) in (**b**), $\varepsilon_{\mathrm{deriv}}^{\prime \prime }$(f) in (**c**), and M″(f) in (**d**). Solid lines are fitting curves. In (**c**,**d**), individual relaxations are shown as dashed and dotted lines. The solid line in (**d**) is conductivity relaxation (see text for details). The arrows in (**c**,**d**) indicate the main peak position.

The best fitting of the data were achieved assuming the contribution of two relaxation processes (main peak and kink at high frequencies), which were modeled by two terms, such as Equation (3), and of electrode polarization (linear increase at low frequencies), which was modeled by a power law function (Af^-s^). Figure 6c and Figure 7c show a representative fitting of $\varepsilon_{\mathrm{deriv}}^{\prime \prime }$ data at 218 K for [N_8881_]Lev and [P_8881_]Lev, respectively. Individual contributions of relaxation processes are shown as dashed and dotted lines in these plots.

For both IL systems under investigation, the main relaxation process is described by a Cole–Davidson (C–D) function (H–N with α = 1) with a b shape parameter equal to 0.4, while Δε of the relaxation decreases from 8.1 to 5.8 (208 to 263 K) and from 9.4 to 5.4 (198 to 263 K) for [N_8881_]Lev and [P_8881_]Lev, respectively. The contribution, observed as a kink at high frequencies, was modeled by a Cole–Cole (C–C) function with a α_CC_ parameter close to 0.3. The latter process is ascribed to secondary relaxations observed in ILs [26,54,55] and will not be discussed further here. However, from Figure 6c and Figure 7c, it is obvious that the secondary dielectric relaxation significantly influences the dielectric response up to 0.5 KHz, and 5 KHz for the [N_8881_]Lev and [P_8881_]Lev ILs, respectively. Dielectric data in all the formalisms can be reproduced assuming the contributions of the relaxation processes obtained by fitting of the $\varepsilon_{\mathrm{deriv}}^{\prime \prime }$ and the dc conductivity. So, dielectric data in different representations (ε′, ε″, $\varepsilon_{\mathrm{deriv}}^{\prime \prime }$, M″) at 218 K for [N_8881_]Lev and [P_8881_]Lev are shown in Figure 6 and Figure 7, respectively. The parameters obtained from the fitting of $\varepsilon_{\mathrm{deriv}}^{\prime \prime }$ data were used to reproduce data in all other representations. For the reproduction of data in different formalisms, additional terms were used. For the reproduction of ε′ (f) (Figure 6a and Figure 7a), the instantaneous permittivity, ε_∞_, for ε″ (f) (Figure 6b and Figure 7b), conductivity term ($\frac{\sigma_{0}}{\varepsilon_{0}2\pi f}$), and for M″(f) (Figure 6d and Figure 7d), both ε_∞_ and the conductivity term were added. The values used are those obtained directly from dielectric data. For both ILs under investigation, the dielectric data are perfectly described in all the representations assuming the contributions of two relaxation processes and of dc conductivity as shown in Figure 6 and Figure 7.

According to this fitting procedure, the broad peak in logM″–logf plots is due to the overlapping of two different mechanisms, as shown in Figure 6d and Figure 7d. The high frequency mechanism corresponds to the main dielectric relaxation in the $\varepsilon_{\mathrm{deriv}}^{\prime \prime }$ representation while the low frequency is the conductivity relaxation mechanism. These two mechanisms are essentially different although both are due to mobile ions. The main dielectric relaxation is a polarization process due to short range ions motion while the second one is the conductivity relaxation mechanism related to the dc conductivity via the relation $\sigma_{0}=\varepsilon_{0}\varepsilon_{s}2{\pi f}_{Μ}$, where f_M_ is the lower frequencies peak in the M″ representation.

**Figure 8:**
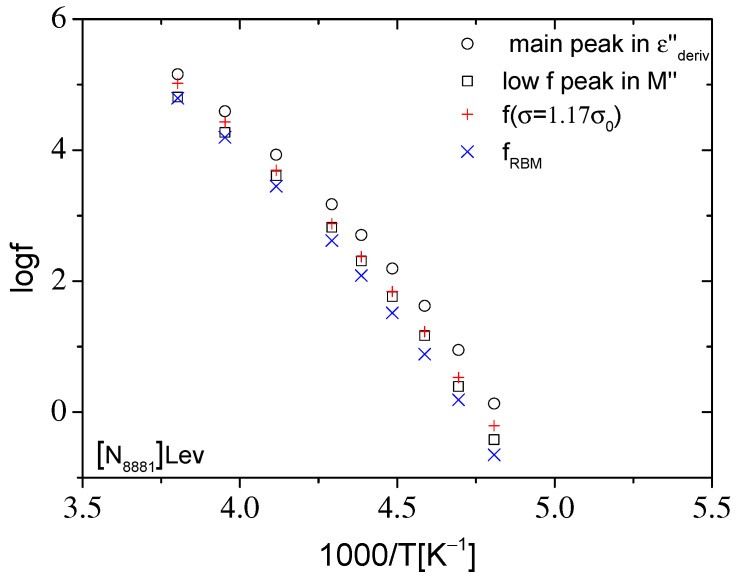
Arrhenius diagram of the characteristic frequencies of the main peak in $\varepsilon_{\mathrm{deriv}}^{\prime \prime }$ and of the low frequency peak in M″, as well as of the frequency at which σ′ = 1.17σ_0_ and of the characteristic frequency fRBM obtained by fitting the expression of the original RBM to σ′(f) data for [N8881]Lev presented in Figure 4.

**Figure 9:**
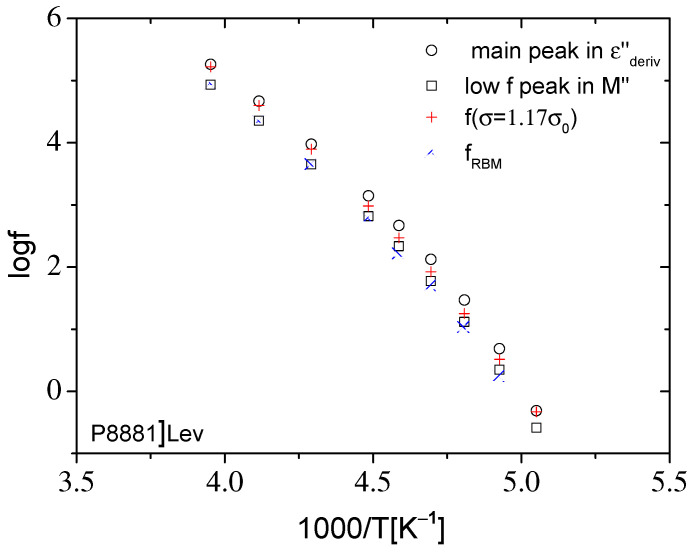
Arrhenius diagram of the characteristic frequencies of the main peak in $\varepsilon_{\mathrm{deriv}}^{\prime \prime }$ and of the low frequency peak in M″, as well as of the frequency at which σ′ = 1.17σ_0_ and of the characteristic frequency f_RBM_ obtained by fitting the expression of the original RBM to σ′(f) data for [P_8881_]Lev presented in Figure 5.

In order to compare the time scale of the relaxations obtained by fitting the dielectric data, the Arrhenius diagram of the characteristic peak frequencies f_ε_ of the main dielectric relaxation in the $\varepsilon_{\mathrm{deriv}}^{\prime \prime }$ representation and the characteristic frequency f_M_ of the conductivity relaxation in M″ are presented in Figure 8 and Figure 9. In the same figures, the characteristic frequencies of the RBM model, f_RBM_, at which σ′ = 1.17σ_0_ (from the experimental data) and the fitting processes, are also included. The unsatisfactory applicability of the RBM model to ionic liquids studied in the present work, even in the dc–ac transition region, is shown by the difference in f_RBM_ frequency values as calculated from the experimental data (σ′ = 1.17σ_0_) and the fitting processes (Figure 8 and Figure 9). The characteristic frequency, f_RBM_, as graphically estimated from σ′–f plots, always takes values between the characteristic frequencies f_ε_ and f_M_ of both ILs. According to the Arrhenius plots of Figure 8 and Figure 9, the relative difference of the characteristic frequencies f_ε_ and f_M_ is higher for [N_8881_]Lev, at each temperature. Moreover, the characteristic frequencies f_ε_ and f_M_ of [P_8881_]Lev are higher than the respective ones of [N_8881_]Lev, which means faster mechanisms.

While the frequency f_M_ is related to the dc conductivity via the relation $\sigma_{0}=\varepsilon_{o}\varepsilon_{s}2{\pi f}_{Μ}$, the frequency f_ε_ of the main dielectric relaxation related also to the dc conductivity via the well-known BNN relation $\sigma_{0}={p\varepsilon }_{o}\Delta \varepsilon 2{\pi f}_{\varepsilon }$, where Δε is the contribution of the main dielectric relaxation in ε’ [56]. The coefficient p usually takes values close and around unity, p≈1 [57]. Therefore, $f_{M}/f_{\varepsilon }\approx \Delta \varepsilon /\left(\Delta \varepsilon +\varepsilon_{\infty }\right)$ and, hence, f_M_ < f_ε_. So, the distances of the characteristic frequencies f_ε_ and f_M_ depend macroscopically on the parameters Δε and the unrelated to ionic motion instantaneous permittivity $\varepsilon_{\infty }$. Microscopically, in the framework of IC structures [58], a possible scenario that could explain the dielectric response of both ionic liquids in Arrhenius plots of Figure 8 and Figure 9 are as follows. Because the stimulus in dielectric relaxation spectroscopy is the alternating electric field, E, the concept of signals period T = 1/f is useful to better perceive the overall dielectric response. The main dielectric relaxation, as detected in ε″ representation, could be a result of the dipolar moments induced by the relative displacements of ions in IC structures by the application of the ac electric field E. Stronger ionic interactions in IC imply more difficult displacements of ions, so a longer duration of E (higher period or lower frequency) is required to complete the polarization mechanism and reach the peak f_ε_. Therefore, stronger ionic interaction means a lower value of f_ε_ and as a consequence, a lower value of f_M_ ($f_{M}<f_{\varepsilon }$) and, hence, lower dc conductivity, a behavior that characterizes the experimental data of [N_8881_]Lev IL. This is in accordance with a recent work [48]. The viscosity values and the related activation energy of both ILs [48], suggest that [N_8881_]Lev has stronger interactions between anion–cation pairs than [P_8881_]Lev. The higher the viscosity and the activation energy, the stronger the ionic interactions.

The conductivity relaxation time is related to the characteristic frequency f_M_, which appears below f_ε_, as shown previously. The relative distance of these two characteristic frequencies depends on how strong the ionic interactions are in the IC structures. Stronger interactions imply that a longer duration of E (higher period or lower frequency) is required to assist the ions to escape from the coulombic cage in IC structures, and then move at a longer distance, giving rise to the conductivity relaxation mechanism as detected in the M″ representation. Therefore, a larger relative difference between the characteristic frequencies f_ε_ and f_M_ indicates stronger ionic interactions, and this behavior characterizes [N_8881_]Lev, as discussed previously. Both characteristic frequencies f_RBM_ and f_M_ are related to the conduction mechanism. According to the Arrhenius diagrams in Figure 8 and Figure 9, f_RBM_ (from σ′ = 1.17σ_0_) is systematically higher than f_M_ and lower than f_ε_, $f_{M}<f_{\mathrm{RBM}}<f_{\varepsilon }$. While f_RBM_ is related microscopically to the ion hopping rate of the largest energy barrier according to the RBM model, f_M_ is directly connected to the macroscopic parameters σ_0_ and ε_s_, which are measurable quantities.

## 3. Materials and Methods

### 3.1. Materials

Trioctylmethylammonium and trioctylmethylphosphonium methylcarbonate methanol solutions were purchased by Proionic GmbH (Raaba-Grambach, Austria). Levulinic acid was provided by Alfa Aesar (Haverhill, MA, USA).

#### Synthesis of [N_8881_]Lev and [P_8881_]Lev

Trioctylmethylammonium levulinate [N_8881_]Lev and trioctylmethylphosphonium levulinate [P_8881_]Lev were synthetized from methylcarbonate precursors following a previously reported procedure [15]. ^1^H and ^13^C NMR, and FTIR spectra, were in agreement with those reported.

### 3.2. Methods

A broadband dielectric spectroscopy (BDS) technique in a broad frequency 10^−1^–10^6^ Hz and temperature 173–333 K range was used to study the ILs. Measurements were performed using an Alpha-A analyzer combined with a Novocool temperature controller, both provided by Novocontrol. The capacitor was prepared by placing ionic liquids between two parallel gold-plated flat electrodes, 20 mm in diameter. The distance between the electrodes was kept constant at 50 μm using silica spacers. Since the transport properties of ILs have been found to be significantly affected even by low water contamination [59], prior to the measurements, the samples were kept at 353 K for 24 h in a vacuum oven. The two ILs analyzed in this work belonged to the same batch of samples studied in the previous work on levulinate-based ILs [48] where the results of Karl Fischer titrations for [N_8881_]Lev and [P_8881_]Lev were 88 and 92 ppm, respectively. The samples were dried at 333 K for 12 h in vacuum before the analysis. Considering that [N_8881_]Lev and [P_8881_]Lev are hydrophobic ILs and that our drying procedure is similar, similar values of water contents can possibly be assumed. A voltage amplitude equal to 0.1 V was used.

## 4. Conclusions

Tetraalkylammonium and its phosphonium homolog as cations were combined with the levulinate anion to form the IL systems studied here. Ionic conductivity and dynamics in these systems were studied by employing the broadband dielectric spectroscopy technique in a broad frequency and temperature range. Between the systems studied here, higher values of ionic conductivity and ion mobility were found for tetraalkylphosphonium-based IL compared to its ammonium homolog in accordance with the lower viscosity found for the former. No significant differences between the systems were found in ion association. A comparison of the systems studied here, with corresponding systems having NTf_2_ as anion, revealed that the levulinate anion used in the present study results in stronger interactions and ion association, and as a consequence to lower values of room temperature conductivity. The analysis presented here demonstrates that the original random barrier model does not describe well the conductivity response, especially in the higher frequency range. The broad low frequency peak in electric modulus representation consists of two overlapping contributions. The slower process is related to the conduction mechanism while the faster process corresponds to the polarization mechanism of the main dielectric relaxation in the complex dielectric permittivity representation. Stronger ionic interactions were found to lead to a slower conductivity relaxation mechanism, which means a lower dc conductivity value and vice versa.

## Figures and Tables

**Table 1:** VFT parameters obtained from fitting of conductivity for [N_8881_]Lev and [P_8881_]Lev.

VTF σ_0_ (T)	[P_8881_]Lev	[N_8881_]Lev
σ_∞_ [S/cm]	1.11	1.92
B	1608	1845
D	11.7	13.2
T_0_ [K]	138	140
m	66	61

## Data Availability

Data supporting this article are available from the corresponding author upon reasonable request.

## References

[1] Tsunashima K., Sugiya M. (2007). Physical and electrochemical properties of low-viscosity phosphonium ionic liquids as potential electrolytes. Electrochem. Commun..

[2] Seki S., Hayamizu K., Tsuzuki S., Fujii K., Umebayashi Y., Mitsugi T., Kobayashi T., Ohno Y., Kobayashi Y., Mita Y. (2009). Relationships between center atom species (n, p) and ionic conductivity, viscosity, density, self-diffusion coefficient of quaternary cation room-temperature ionic liquids. Phys. Chem. Chem. Phys..

[3] Shirota H., Fukazawa H., Fujisawa T., Wishart J. (2010). Heavy atom substitution effects in non-aromatic ionic liquids: Ultrafast dynamics and physical properties. J. Phys. Chem. B.

[4] Carvalho P.J., Ventura S.P., Batista M.L., Schroeder B., Gonçalves F., Esperança J., Mutelet F., Coutinho J.A. (2014). Understanding the impact of the central atom on the ionic liquid behavior: Phosphonium vs ammonium cations. J. Chem. Phys..

[5] Philippi F., Rauber D., Kuttich B., Kraus T., Kay C.W., Hempelmann R., Hunt P.A., Welton T. (2020). Ether functionalisation, ion conformation and the optimisation of macroscopic properties in ionic liquids. Phys. Chem. Chem. Phys..

[6] Hulsbosch J., Vos D.E., Binnemans K., Ameloot R. (2016). Biobased ionic liquids: Solvents for a green processing industry?. ACS Sustain. Chem. Eng..

[7] Seitkalieva M.M., Vavina A.V., Posvyatenko A., Egorova K.S., Kashin A., Gordeev E., Strukova E.N., Romashov L.V., Ananikov V. (2021). Biomass-derived ionic liquids based on a 5-HMF platform chemical: Synthesis, characterization, biological activity, and tunable interactions at the molecular level. ACS Sustain. Chem. Eng..

[8] Cheng X., Liu Y., Wang K., Yu H., Yu S., Liu S. (2021). High-efficient conversion of cellulose to levulinic acid catalyzed via functional brønstedlewis acidic ionic liquids. Catal. Lett..

[9] Shahriari S., Tomé L., Araújo J.M., Rebelo L.P., Coutinho J.A., Marrucho I.M., Freire M. (2013). Aqueous biphasic systems: A benign route using cholinium-based ionic liquids. RSC Adv..

[10] Wagner V., Schulz P., Wasserscheid P. (2014). Asymmetric hydrogenation catalysis via ion-pairing in chiral ionic liquids. J. Mol. Liq..

[11] Stevanovic S., Podgorsek A., Moura L., Santini C.C., Padua A.A.H., Gomes M.F. (2013). Absorption of carbon dioxide by ionic liquids with carboxylate anions. Int. J. Greenh. Gas Control.

[12] Avila J., Lepre L.F., Santini C.C., Tiano M., Denis-Quanquin S., Szeto K.C., Padua A.A.H., Gomes M.C. (2021). High-performance porous ionic liquids for low-pressure CO_2_ capture. Angew. Chem. Int. Ed..

[13] Boissou F.T., Muehlbauer A., Vigier K.d., Leclercq L., Kunz W., Marinkovic S., Estrine B., Nardello-Rataj V., Jérôme F. (2014). Transition of cellulose crystalline structure in biodegradable mixtures of renewably-sourced levulinate alkyl ammonium ionic liquids, γ-valerolactone and water. Green Chem..

[14] Becherini S., Mezzetta A., Chiappe C., Guazzelli L. (2019). Levulinate amidinium protic ionic liquids (PILs) as suitable media for the dissolution and levulination of cellulose. New J. Chem..

[15] Mezzetta A., Becherini S., Pretti C., Monni G., Casu V., Chiappe C., Guazzelli L. (2019). Insights into the levulinate-based ionic liquid class: Synthesis, cellulose dissolution evaluation and ecotoxicity assessment. New J. Chem..

[16] He F., Chen J., Gong Z., Xu Q., Yue W., Xie H. (2021). Dissolution pretreatment of cellulose by using levulinic acid-based protic ionic liquids towards enhanced enzymatic hydrolysis. Carbohydr. Polym..

[17] Sangoro J.R., Serghei A., Naumov S., Galvosas P., Kaerger J., Wespe C., Bordusa F., Kremer F. (2008). Charge transport and mass transport in imidazolium-based ionic liquids. Phys. Rev. E.

[18] Sangoro J.R., Iacob C., Serghei A., Friedrich C., Kremer F. (2009). Universal scaling of charge transport in glass-forming ionic liquids. Phys. Chem. Chem. Phys..

[19] Krause C., Sangoro J.R., Iacob C., Kremer F. (2009). Charge transport and dipolar relaxations in imidazolium-based ionic liquids. J. Phys. Chem. B.

[20] Sippel P., Krohns S., Reuter D., Lunkenheimer P., Loidl A. (2018). Importance of reorientational dynamics for the charge transport in ionic liquids. Phys. Rev. E.

[21] Thoms E., Sippel P., Reuter D., Weiß M., Loidl A., Krohns S. (2017). Dielectric study on mixtures of ionic liquids. Sci. Rep..

[22] Pabst F., Gabriel J., Weigl P., Blochowicz T. (2017). Molecular dynamics of supercooled ionic liquids studied by light scattering and dielectric spectroscopy. Chem. Phys..

[23] Ito N., Huang W., Richert R. (2006). Dynamics of a supercooled ionic liquid studied by optical and dielectric spectroscopy. J. Phys. Chem. B.

[24] Griffin P.J., Agapov A.L., Kisliuk A., Sun X.-G., Dai S., Novikov V.N., Sokolov A.P. (2011). Decoupling charge transport from the structural dynamics in room temperature ionic liquids. J. Chem. Phys..

[25] Griffin P.J., Holt A.P., Wang Y., Novikov V.N., Sangoro J.R., Kremer F., Sokolov A.P. (2014). Interplay between hydrophobic aggregation and charge transport in the ionic liquid methyltrioctylammonium bis(trifluoromethylsulfonyl)imide. J. Phys. Chem. B.

[26] Griffin P.J., Holt A.P., Tsunashima K., Sangoro J.R., Kremer F., Sokolov A.P. (2015). Ion transport and structural dynamics in homologous ammonium and phosphonium-based room temperature ionic liquids. J. Chem. Phys..

[27] Pabst F., Wojnarowska Z., Paluch M., Blochowicz T. (2021). On the temperature and pressure dependence of dielectric relaxation processes in ionic liquids. Phys. Chem. Chem. Phys..

[28] Cosby T., Sangoro J., Kremer F., Paluch M. (2016). Rotational and Translational Diffusion in Ionic Liquids. Dielectric Properties of Ionic Liquids.

[29] Griffin P.J., Agapov A.L., Sokolov A.P. (2012). Translation-rotation decoupling and nonexponentiality in room temperature ionic liquids. Phys. Rev. E.

[30] Dyre J.C. (1985). A simple model of ac hopping conductivity in disordered solids. Phys. Lett. A.

[31] Schrøder T.B., Dyre J.C. (2008). ac hopping conduction at extreme disorder takes place on the percolating cluster. Phys. Rev. Lett..

[32] Dyre J.C. (1988). The random free-energy barrier model for ac conduction in disordered solids. J. Appl. Phys..

[33] Gainaru C., Stacy E.W., Bocharova V., Gobet M., Holt A.P., Saito T., Greenbaum S., Sokolov A.P. (2016). Mechanism of conductivity relaxation in liquid and polymeric electrolytes: Direct link between conductivity and diffusivity. J. Phys. Chem. B.

[34] Silva W., Zanatta M., Ferreira A.S., Corvo M.C., Cabrita E.J. (2020). Revisiting ionic liquid structure-property relationship: A critical analysis. Int. J. Mol. Sci..

[35] Xu W., Cooper E.I., Angell C.A. (2003). Ionic liquids: Ion mobilities, glass temperatures, and fragilities. J. Phys. Chem. B.

[36] Cosby T., Vicars Z., Mapesa E.U., Tsunashima K., Sangoro J. (2017). Charge transport and dipolar relaxations in phosphonium-based ionic liquids. J. Chem. Phys..

[37] Cosby T., Vicars Z., Heres M., Tsunashima K., Sangoro J. (2018). Dynamic and structural evidence of mesoscopic aggregation in phosphonium ionic liquids. J. Chem. Phys..

[38] Griffin P.J., Wang Y., Holt A.P., Sokolov A.P. (2016). Communication: Influence of nanophase segregation on ion transport in room temperature ionic liquids. J. Chem. Phys..

[39] Wuebbenhorst M., van Turnhout J. (2002). Analysis of complex dielectric spectra. i. one-dimensional derivative techniques and three-dimensional modelling. J. Non-Cryst. Solids.

[40] Macdonald J.R. (2009). Comments on the electric modulus formalism model and superior alternatives to it for the analysis of the frequency response of ionic conductors. J. Phys. Chem. Solids.

[41] Lunkenheimer P., Bobnar V., Pronin A.V., Ritus A.I., Volkov A.A., Loidl A. (2002). Origin of apparent colossal dielectric constants. Phys. Rev. B.

[42] MacDonald J.R. (1987). Comparison and discussion of some theories of the equilibrium electrical double layer in liquid electrolytes. J. Electroanal. Chem. Interfacial Electrochem..

[43] Serghei A., Tress M., Sangoro J.R., Kremer F. (2009). Electrode polarization and charge transport at solid interfaces. Phys. Rev. B.

[44] Emmert S., Wolf M., Gulich R., Krohns S., Kastner S., Lunkenheimer P., Loid A.l. (2011). Electrode polarization effects in broadband dielectric spectroscopy. Eur. Phys. J. B.

[45] Angell C.A., Ngai K.L., Wright G.B. (1985). Strong and fragile liquids. Relaxations in Complex Systems.

[46] Boehmer R., Ngai K.L., Angell C.A., Plazek D.J. (1993). Nonexponential relaxations in strong and fragile glass formers. J. Chem. Phys..

[47] Sippel P., Lunkenheimer P., Krohns S., Thoms E., Loidl A. (2015). Importance of liquid fragility for energy applications of ionic liquids. Sci. Rep..

[48] Mero A., Guglielmero L., Andrea F.D., Pomelli C.S., Guazzelli L., Koutsoumpos S., Tsonos G., Stavrakas I., Moutzouris K., Mezzetta A. (2022). Influence of the cation partner on levulinate ionic liquids properties. J. Mol. Liq..

[49] Angell C.A., Byrne N., Belieres J.-P. (2007). Parallel developments in aprotic and protic ionic liquids: Physical chemistry and applications. Acc. Chem. Res..

[50] Fraser K.J., Izgorodina E.I., Forsyth M., Scott J.L., MacFarlane D.R. (2007). Liquids intermediate between molecular and ionic liquids: Liquid ion pairs?. Chem. Commun..

[51] MacFarlane D.R., Forsyth M., Izgorodina E.I., Abbott A.P., Annat G., Fraser K. (2009). On the concept of ionicity in ionic liquids. Phys. Chem. Chem. Phys..

[52] Musia M., Bair S., Cheng S., Wojnarowska Z., Paluch M. (2021). Fractional walden rule for aprotic ionic liquids: Experimental verification over a wide range of temperatures and pressures. J. Mol. Liq..

[53] Havriliak S., Negami S. (1967). A complex plane representation of dielectric and mechanical relaxation processes in some polymers. Polymer.

[54] Rivera A., Roessler E.A. (2006). Evidence of secondary relaxations in the dielectric spectra of ionic liquids. Phys. Rev. B.

[55] Rivera A., Brodin A., Pugachev A., Roessler E.A. (2007). Orientational and translational dynamics in room temperature ionic liquids. J. Chem. Phys..

[56] Namikawa H. (1975). Characterization of the diffusion process in oxide glasses based on the correlation between electric conduction and dielectric relaxation. J. Non-Cryst. Solids.

[57] Tsonos C., Kanapitsas A., Kechriniotis A., Petropoulos N. (2012). AC and DC conductivity correlation: The coefficient of Barton-Nakajima-Namikawa relation. J. Non-Cryst. Solids.

[58] Stacy E.W., Gainaru C.P., Gobet M., Wojnarowska Z., Bocharova V., Greenbaum S.G., Sokolov A.P. (2018). Fundamental limitations of ionic conductivity in polymerized ionic liquids. Macromolecules.

[59] Ausín D., Parajó J.J., Trenzado J.L., Varela L.M., Cabeza O., Segade L. (2021). Influence of small quantities of water on the physical properties of alkylammonium nitrate ionic liquids. Int. J. Mol. Sci..

